# Research on Automated Modeling Technology of Sluice Gate Project Based on BIM Technology

**DOI:** 10.3390/s24165114

**Published:** 2024-08-07

**Authors:** Jiedeerbieke Madiniyeti, Qiujing Zhou, Huijun Qi, Yang Chao, Yu Zhang

**Affiliations:** 1 College of Hydraulic and Civil Engineering, Xinjiang Agricultural University, Urumqi 830052, China; jedel@xjau.edu.cn (J.M.); qihuijun@hhu.edu.cn (H.Q.); 2 Key Laboratory of River Basin Digital Twinning of Ministry of Water Resources, Beijing 100048, China; 3 College of Water Conservancy and Hydropower Engineering, Hohai University, Nanjing 210098, China; chaoyangccc@hhu.edu.cn (Y.C.); hhuhszy@outlook.com (Y.Z.)

**Keywords:** sluice gate engineering, Revit API, parameterized family, template library

## Abstract

In order to improve the efficiency and accuracy of the modeling and design work of the sluice gate project, this paper proposes an automatic generation template of the sluice gate project with customized semantics and project layout scheme, aiming at realizing the rapid assembling of all kinds of components of the sluice gate project. In the construction process, this paper first starts from basic parametric modeling and proposes constraints as the basis of modeling. Subsequently, a template library framework is developed based on the constraints to ensure that the generated templates have a high degree of standardization and consistency. Finally, an efficient and flexible template library is successfully constructed by using the customized classes and functions of Revit API, which provides powerful technical support for the modeling and design work of sluice gate engineering. This achievement helps to promote the informationization and intelligent development of the water conservancy engineering industry, and its versatility and scalability also make it have a wide range of application prospects in other water conservancy engineering fields.

## 1. Introduction

With the rapid development of information technology and the increasingly complicated construction of water conservancy projects, the traditional design, construction, and management of water conservancy projects have been difficult to meet the actual needs of modern water conservancy projects. In this context, BIM (building information modeling) technology came into being and has shown great application potential in the field of hydraulic engineering. The core advantage of BIM technology is that it integrates a three-dimensional digital model of multi-stage information such as design, construction, and operation and maintenance. This model not only accurately expresses the physical form of hydraulic engineering, but also deeply integrates its functional characteristics, material properties, construction processes, and even operation and maintenance management and other all-round information. For water conservancy projects, BIM of information integration shows great potential and value, which promotes the improvement in design efficiency, the optimization of construction management, and the improvement in cooperation ability [1,2,3]. Although in water conservancy projects, BIM technology has shown some potential for application, it also faces a series of challenges and limitations, such as information exchange and sharing between multiple specialties, which cannot ensure the consistency and accuracy of data. Complex topography and dynamic water flow conditions increase the difficulty and accuracy of model construction. In addition, the non-uniformity of data interaction standards also hinders the smooth flow and utilization of information [4,5]. Due to the complexity and specificity of water conservancy projects, it often takes a lot of time and effort to optimize and integrate the model building process. Individual parametric models are relatively simple to build, but the real challenge lies in the later integration process. The connection between each independent model is intricate and complex, and it is necessary to accurately match the connection relationship between terrain and structure. In addition, software operation, cutting, merging, and other fine adjustments not only require superior skills but also bring a heavy workload, as each step needs to be meticulous to ensure that the overall model exhibits seamless and efficient operation. This paper focuses on considering the problems and solutions encountered in the parametric modeling and integration process of complex buildings, the core reason for which is that the application of BIM technology or the practice of the digital twin concept cannot be separated from the most basic model parameterization and accuracy. Parametric modeling is the core of BIM technology which defines components and elements in a parametric way and establishes the relationship between parameters and models [6,7,8]. The fusion of parametric models and parameter rationality, which together ensure the efficient and accurate combination of components to form a complete and consistent model, is also an important aspect in the application of BIM technology.

The sluice gate project is a class of low-head hydraulic structures built on rivers, lakes, channels, seawalls, or reservoir banks [9,10]. Multiple studies have shown that it is now possible to create parametric drive models of buildings related to sluice gate engineering in Revit 2020 software and effectively perform complete stitching of resources and models [11,12,13,14,15,16]. The earliest study by Ren et al. [11] proposed a system of BIM integrated into a design solution by taking a small pump gate project as an example, applying BIM technology to realize the modules of modeling, collaboration, collision, drawings, and reports in the design stage. Jiang et al. [12] performed parametric modeling of a sluice gate, ad extraction of the model’s quantities, and preparation of project estimates by means of secondary development, which further confirmed the practicality and applicability of BIM technology. Jiang et al. [13] showed that combined with the characteristics of different cross-section types of farmland channels, the Revit API interface is used to carry out the secondary development of Revit software, and intelligent modeling and three-dimensional reinforcement methods for farmland channels are proposed. Yuan et al. [14] analyzed the deficiencies in the construction safety process using BIM technology and a safety knowledge base for several hydraulic buildings. In addition, there are studies on similar buildings, such as Zhang et al. [17]. A new geological model building method was proposed by adopting a topological geometry modeling strategy and using parametric modeling. Geng et al. [18] established a water conservancy project construction progress management system based on building information modeling and GIS, which provides a three-dimensional dynamic visualization environment for water conservancy projects, and fine control of a water conservancy project’s volume and progress plan management. Zhang et al. [19], with the combination of BIM technology and WebGIS, proposed an extended IFC export method for hydropower projects and a data exchange method between extended IFC and batch 3D Tiles, and the validity of the method was verified through a case of design modification of a crushed concrete gravity dam. Gong et al. [20] developed an automated assembly program that can be used for tunnel tube sheets, which also involves different levels of parametric framework construction and application. From most of the literature, it can be seen that at present, the fully parametric sluice model is basically solved, and even the accuracy of the model has reached a high degree, but the research on the accurate construction and automated assembly for the complex constraint relationship between models is still insufficient; complex buildings still take a lot of time to assemble, and some of the models may lose the comparative advantage of efficiency compared to two-dimensional modeling due to collision problems. In particular, how to efficiently and accurately define the physical, geometric, and functional constraints between different model components in sluice gate engineering, as well as how to realize the intelligent and automated assembly and integration of these models, is still a challenge to be solved. Therefore, it is necessary to study the efficient integration method of the model and explore new ways to effectively improve modeling efficiency and quality. Taking a sluice gate project as a case study, this paper aims to reveal the parametric modeling and efficient integration of the sluice gate model, and with the help of Revit’s secondary development means, it further proposes the parameter semantics of each component and an automation arrangement scheme to simplify the modeling process and improve the efficiency of modeling, so as to meet the urgent needs of the water conservancy engineering field for efficient and accurate modeling.

## 2. Revit Secondary Development Technology

### 2.1. Revit API Toolkit

Revit software is a series of software designed for building information modeling (BIM) developed by Autodesk, through which Revit can effectively solve the problem of model-driven design and improve the efficiency and quality of design [7,21]. In order to meet specific project requirements, the functionality of Revit software needs to be extended and customized, usually by using Revit API for in-depth development or writing Revit built-in scripts for automation. Also, to simplify repetitive tasks, the Revit macro editor is utilized. The majority of Revit external applications in use today are installed on the platform through a variety of secondary development routes that provide a wide range of customization options and enhancements [22,23]. Among them, Revit API, as the most direct and powerful development tool, provides developers with the ability to interact with Revit’s core functionality in depth and is the preferred method to achieve customized functionality extensions [24,25]. Revit API contains a rich set of namespaces, classes, and their functions and attributes, which are resources that provide strong support for secondary development and greatly reduce the difficulty of development, enabling developers to extend and customize the functionality of Revit software more efficiently. In the development of the template library, a variety of Revit API built-in functions and classes are used, such as using the methods in the Document class to create, delete, or modify elements, and using the Transaction class to encapsulate changes to ensure that they are executed in a single transaction. For view manipulation, the View class and its subclasses are used to obtain, create, or modify views. While built-in functions can address the implementation of basic functionality, they are not designed to be “plug and play” solutions for specific applications or template libraries. Therefore, when developing template libraries for automated family assembly, custom classes or functions are often needed to meet specific functionality requirements to support automated family alignment and accurate model placement. Table 1 shows the main Revit API custom functions or classes used to build template libraries.

Revit API provides two ways to extend its functionality: one is the IExternal Command interface, which is used to define external commands, in which the user clicks on the added command button to launch the corresponding command generated by the secondary development; and the other way, the IExternal Application interface, is used to create an external application, i.e., to add menus or toolbars, which is automatically executed when Revit starts and shuts down. Since IExternal Application provides a higher level of extensibility, it is common to use IExternal Application to overload OnStartup( ) and OnShutdown( ) functions to realize the development of functions.

### 2.2. Interface Development

**Figure 1:**
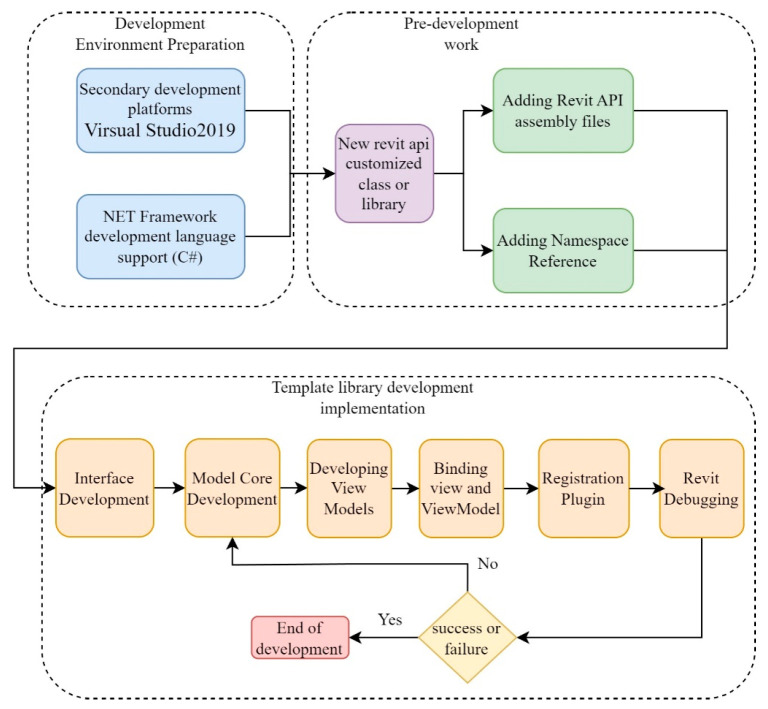
Revit software customization development process based on MVVM pattern.

WPF (Windows Presentation Foundation) is a Microsoft graphical presentation system based on the .NET Framework that provides a rich set of controls and layout options that enable developers to easily create beautiful and easy-to-use user interfaces [26,27]. WPF introduces data-driven core concepts such as DataDriven, DataBinding, and the XAML language which significantly separate the user interface (UI) design from the underlying logic. At the same time, WPF utilizes the DirectX underlying interface, its powerful graphics vector-rendering engine compared to the previous generation of CDI/GDI + programming model, to achieve a qualitative leap. Therefore, WPF in the field of interface development has occupied a significant position. The MVVM design pattern is a software architecture pattern for developing GUI applications that separates the user interface (View) from the business logic (Model) and introduces an intermediate layer, called ViewModel, to coordinate the interactions between View and Model [13,28]. The MVVM pattern makes full use of the XAML in WPF and the DataBinding features, layered to achieve functional modularity and reduce the coupling between individual modules, so that the program’s architecture is clear and the functionality is clear, while facilitating collaborative team development. When developing a Revit API-based automatic family template library in MVVM mode, the ViewModel layer calls Revit API to realize the interaction with BIM, performs the reading, modification, and updating of BIM model, and ensures that the application logic is seamlessly integrated with the Revit environment. The View layer is responsible for the display and interaction of the user interface through rich interactive elements and collects user input feedback to be processed by the ViewModel layer so as to realize a clear separation of user interactions and business logic. The Model layer manages business data and logic, organizes and manages data, stores core information such as template configuration and parameter settings, and provides solid data support for the template library. The three work together to realize the functional requirements and ensure the effective separation of interface design and business logic. The use of MVVM mode needs to be fully understood and prepared for the development environment and the need to call the Revit API classes or functions to ensure that the development process can efficiently use the API to achieve the operational requirements of BIM. The specific development process is shown in Figure 1.

## 3. Sluice Engineering Template Library Creation

### 3.1. Revit Parametric Design Routine Flow

**Figure 2:**
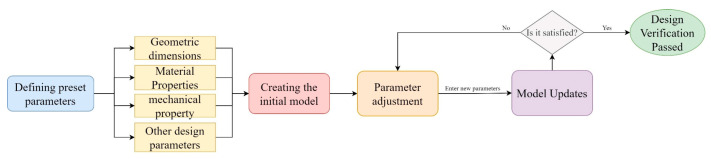
Conceptual flowchart of parametric design.

Parametric design, which is based on a set of predefined parameters to create, modify, and control a design model, represents a more intelligent and efficient way of thinking about design [29,30]. The set parameters can be geometric dimensions, material properties, mechanical properties, etc., which together define the characteristics and behavior of the model. By adjusting the parameters, the model can be rapidly iterated and optimized to meet different design requirements and constraints. In parametric design, the dimensional parameter-driven process is a core concept implying precise control by tuning specific dimensional parameters [31,32]. Figure 2 shows the conceptual flowchart of conventional parametric design, which shows that the first step is to preset the basic parameter framework of the model and then achieve the flexible modification of model dimensions and attributes by continuously adjusting these parameters. This process is repeated until the model fully meets the requirements, which also reflects the advantages of efficient iteration and precise control of parametric design. For the modeling of Revit-driven models of conventional hydraulic buildings, one can refer to a study written by the authors [16]. In this study, the core of parametric modeling focuses mainly on the 3D semantics of sluice gate engineering components and the definition of their constraints.

### 3.2. Definition of Constraints

**Figure 3:**
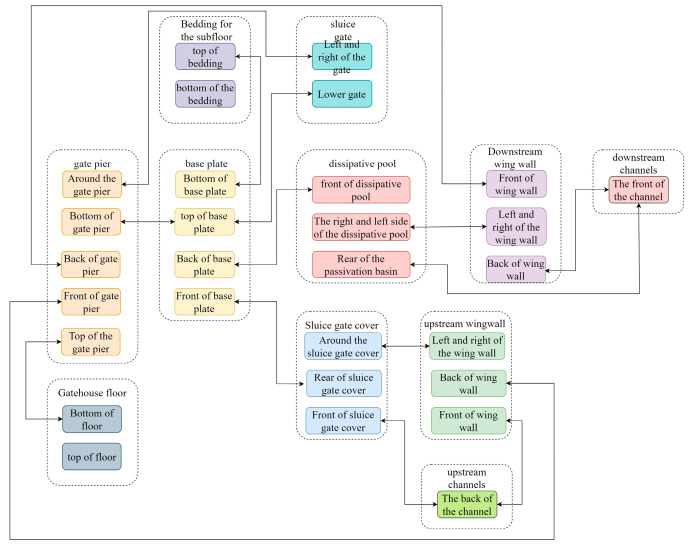
Schematic diagram of main constraints of each member of sluice project.

In the process of parametric modeling of the sluice gate project using Revit, given that the overall model is pieced together by multiple families of components, it is necessary to determine the constraints between the components before building to ensure precise articulation between the components. By determining the constraint relationships, the modeling sequence of the components can be further judged and optimized. Subsequently, models or auxiliary graphics such as geometries, reference planes, and dimensional annotations are created. Next, the key step of model parameterization is carried out by associating the reference plane with the model boundary and closely associating the dimension annotation with the parameters. For example, for the gate pier model, according to the spatial location of the relationship between the components and the need for structural stability, the bottom of the gate pier must be strictly constrained in the elevation of the base plate and the use of fixed constraints must be fixed. Therefore, when building the gate pier model, parameter adjustment of the height of the gate pier should be limited to the upward extension, and its bottom elevation should be kept constant, as any change may affect the accurate position of the base plate. Through the above analysis of the gate pier, it can be argued that the components need to be set between the rigorous articulation relationship in order to ensure the accuracy of model construction while achieving the flexibility of the adjustment of parameterization. Figure 3 is a schematic diagram of the main constraints between the components of the sluice gate project.

Special attention should be paid to the fact that, according to the constraint relationship of each component, in order to facilitate the overall layout and avoid constraint conflicts, it is necessary to build the model from the most constrained component first. The order of building components should not only consider the articulation between components, but also consider the stability and integrity of the overall structure. As shown in Figure 3, the gate pier, as the most constrained component, should be built first. On this basis, the bottom plate and gate can be further parameterized through modeling to ensure that they are smoothly connected with the gate pier. After completing the modeling of the bottom plate and gate, the dissipative pool, cover, wing wall, and other parts are gradually modeled to ensure that the entire model construction process is orderly and efficient.

### 3.3. Custom Semantics and Project Layout Scheme Definition

Before the development of the template library, the original abstract project layout is constructed by humans, using the data storage technology to achieve its concretization, and then the “custom semantics and project layout” is created. Therefore, the essence of the sluice gate project template library is the “custom semantics and project layout plan” for automated modification, saving, and transfer. “Customized semantics and project layout” are based on JSON format, which is used to record component information and layout. In this project, each individual component of the lock project is abstracted into the class TemplateArrangeData, which contains six generic pieces of information, namely name, location, size, whether it is slit or not, load family, and load family type. For specific components, such as base plates, in addition to the generalized data, several pieces of information are included in the dimensions, such as length, thickness, width, bedding thickness, toothed wall thickness, toothed wall angle, etc., with different types of dimensions defined for each component. The parameters of the main setup semantics are shown in Table 2. The semantic representation of specific constructs is as follows:

public class TemplateArrangeData : BaseViewModel

{//Name

public BuiltInCType Name { get; set; }

//Location

public Point Location { get; set; } = new Size(152.36);

//Size

public Size Size { get; set; } = new Size(4000,500,3200,100,300,30,100,300,30);

//HasParting

public bool HasParting { get; set; }

//Family Name

public string Family { get; set; }

FamilySymbol Name.

public string FamilySymbol { get; set; }}.

In the preliminary parametric modeling process, the constraints and articulation conditions between the components were studied in detail, and a set of construction sequences were developed, which are, in order, gate piers, bottom plates, gates, dissipative pools, decks, wing walls, etc. The components were then serialized into JSON files with the file extension “proj”. After these components were set up through the human–computer interface, they were serialized into JSON files with the suffix “proj”. Each project layout plan contains a Name field, which is named by the user when saving. At the same time, the “Data” field records the TemplateArrangeData array and records the content of the project layout.

**Table 2:** Setting of main parameters of each component.

	Parameter 1	Parameter 2	Parameter 3	Parameter 4	Parameter 5	Parameter 6
gate pier	length	height	height	depth of Tank 1	length of Tank 1	distance of Tank 1 from upstream
base plate	height	length	height	thickness	bedding thickness	angle of tooth wall 1
sluice gate	height	height	thickness	border width	rib width	rib thickness
dissipative pool	length	height	thickness	plane diffusion angle	downslope Height	height of tooth wall No 1
wing wall	front width of base plate	thickness of base plate	back width of base plate	wall width	wall height	wall chamfer width

### 3.4. Module Development

Based on the “custom semantics and project layout scheme”, the process of realizing the rapid assembly of the template library can be divided into two steps. In the first step, on the basis of the three-dimensional model of the sluice gate, the three-dimensional semantic model and the plane pre-arrangement design plan are imported to generate the project layout plan file (*.proj), and the second step is to instantiate the model in the project space according to the file and assemble the model of the whole project. The instantiation operation is generally a step-by-step operation, according to the modeler’s modeling habits of step-by-step instantiation, and each step can modify the parameters of the components according to the needs of the gradual assembly of the model. The template library is fully compliant with the “custom semantics and project layout scheme” file, and instantiation is performed silently. In this form, the system automatically reads the semantic information and completes model instantiation according to the preset rules and parameters. The entire process requires no manual intervention from the user, which greatly improves work efficiency. The generated “custom semantics and project layout scheme” file will be saved in the data folder of the platform root directory, which can be modified and reassembled after engineering optimization. Therefore, the function of the template library is mainly centered on four core aspects, which are as follows: (1) Support for floor plan layout pre-design. After defining the data semantics and business logic in the Model layer, it is in the View layer so that the user can carry out preliminary layout planning. (2) Support step-by-step assembly. Define the adjustment parameter mechanism in the Model layer to allow users to modify the parameters of building blocks in each step to meet specific needs. (3) Support silent assembly. Define instantiated interaction logic in the Model layer to provide the automated assembling process. (4) JSON-based persistence of the project layout scheme. Support importing/exporting JSON configuration files to load/save the project layout scheme, which facilitates the sharing and reusing of the design results.

## 4. Project Case

### 4.1. Project Overview

**Figure 4:**
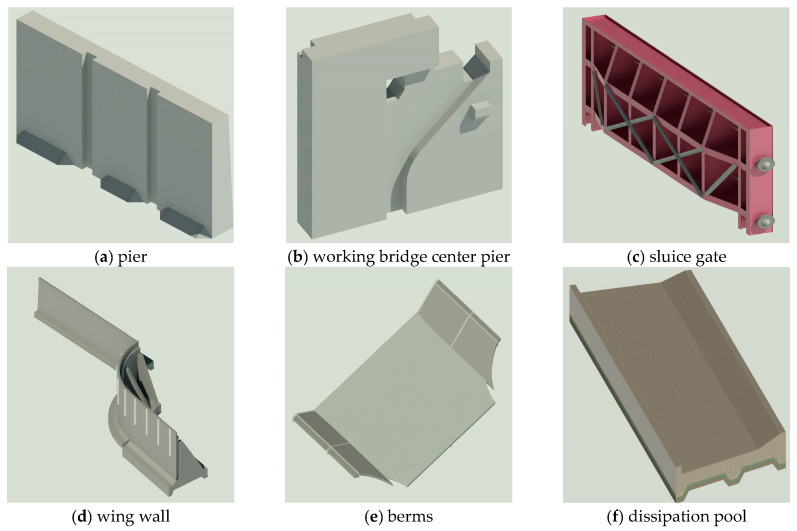
Partially parameterized family models.

The Xingou River Extension and Dredging Project is a project approved by the State Council for the comprehensive improvement in the water environment in the Taihu Lake Basin, and is also one of the key projects for the implementation of the flooding of the basin to the north of the Yangtze River in the Taihu Lake Basin Flood Control Plan. The object of this study is the construction of a sectional gate with two holes of a net width of 12 m and a gate bottom plate with a top elevation of −0.12 m, using a reinforced concrete dock structure, with a rising horizontal plane steel gate. Figure 4 shows the main structural diagram of the sluice gate, which shows the bottom plate and mat structure with toothed walls, and the dissipation pond is also set with multiple toothed walls and is indented on the left and right sides. The upstream wing wall structure has a curved shape, while the upper part of the gate pier is skillfully designed with construction details that are closely connected with the working bridge and the gate chamber; so, the overall structure of the sluice gate is relatively more complicated. The main building level of the gate is level 3, the secondary building is level 4, the peak acceleration of ground vibration is 0.1 g, and the basic intensity of any corresponding earthquake is degree V. 

### 4.2. Parametric Modeling

The sluice gate consists of several components, and in order to ensure that the model can be efficiently spliced and maintain the integrity of the whole structure, the main model is divided into three partitions, namely the gate chamber section, the upstream connecting section, and the downstream connecting section, to create the model. Through the constraint framework system set up in the previous stage, the geometry, reference planes, and parameters were created in detail, and the association between parameters and annotations, reference planes, and models was realized. A total of 42 parameterized custom families were created without counting the system families, and each family was carefully designed and optimized to meet the complex needs of the sluice model, and the specific families created are shown in Table 3. Among them, the construction of the handrail arc wing wall family is relatively complex, as each wing wall family is a combination of the wing wall as well as the main body, handrail array model, triangular angle, trapezoidal tooth chop, wave protection wall, bedding and other ancillary components. The wing wall families use adaptive conventional model templates, and adaptive points can be created by modifying the reference points. The geometry drawn by capturing the adaptive points will produce adaptive components. During the construction process, for model flexibility, the wing wall body is divided into two parts: the base plate and the vertical wall. The base plate is further divided into the front toe plate and the back toe plate, but there is no dividing line in the middle, and the height and width of the divided model are used as the reference variables. The handrail array is a model made up of a single handrail arrayed on a segmentation path and controlled by parameters such as upper and lower bottom widths, width, and height. The other families of models are usually relatively simple to model, and the parameter settings and controls are more intuitive and easier, so they are not described in detail. Figure 4 shows a partially created parametric family model.

### 4.3. Application of the Template Library

**Figure 5:**
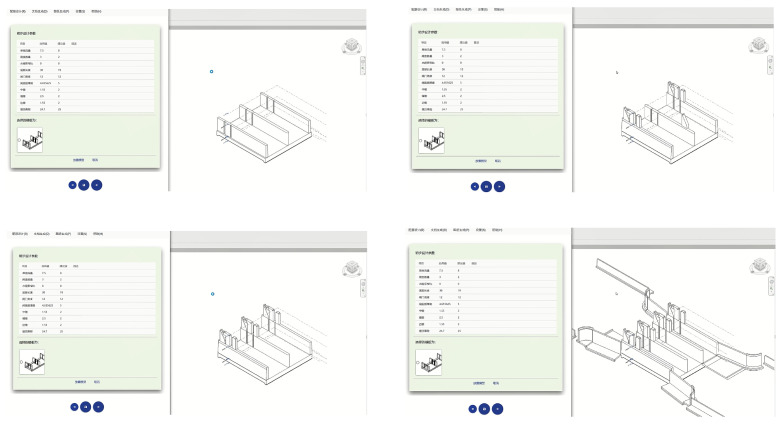
A diagram of the assembling process of the sluice gate.

The template library mainly realizes the extension of the functional area through the external application (IExternal Application) and invokes the external command to display the interactive window for each model parameter input. Therefore, on the basis of the “custom semantics and project layout scheme”, relying on the Visual Studio platform, combined with the NET Framework, using C# programming language, and combined with the above mentioned several custom classes and functions, we can build and extend the functionality of the template library to achieve richer and more efficient family splicing and management. Here, we take two representative complex components, the wing wall and base plate, as our main focus to elaborate on the specific implementation of the template library. TemplateArrangeView is an XAML file of a template layout page which is rendered directly into a template layout view. Based on the MVVM programming model, the view has a unique corresponding view model TemplateArrangeVm, and also contains a custom control DrawingCanvas. The TemplateArrangeVm view model covers the template layout view of the save, import, view, list, delete, add, edit, etc., operations. Therefore, through the cooperative work of TemplateArrangeView and TemplateArrangeVm, it can realize the efficient display of the template layout interface and the user interaction functions. DrawingCanvas, as a custom control in TemplateArrangeView, rewrites the Canvas control in the WPF standard, adds mouse operation functions, and is a special element in View. DrawingCanvas provides important APIs such as ClearCanvas, DeleteVisual, AddVisual, ReDraw, CenterVisuals, and so on, which are available to TemplateArrangeView, and provides powerful dynamic management functions for the TemplateConfigView, which is also a special element in View that can change the river direction and the number of sluice holes, and pre-read the “*.proj” custom project layout file to quickly load and configure the existing template layout. This saves time and effort in setting up the project from scratch. On the basis of the template layout and operation functions, WindowPlaceBottomPlate is used to place window templates, make further changes to labels and view models, and provide an interface for inputting calculation parameters. For bottom plate members, the properties of width and length can be regulated through the PlaceBottomPlateVm view model. PlaceBottomPlateVm is a component of the ViewModel layer. During the instantiation process, IPlaceLockChamberBottomPlate placement logic is used to create elevations (LevelSet) for the bottom plate model, read model parameters entered by the user, locate operations, call the view window, place the model, and perform a series of operations. IPlaceLockChamberBottomPlate and the processing logic of the other classes is similar. For the wing wall component, WindowPlaceWingWall is the xaml file for placing the window of the wing wall. Since the wing wall is constructed by the self-adaptive conventional model, the placing window is a little bit more complicated, but it also provides a human–machine interface for inputting the parameters of the calculation. PlaceWingWallVm is the view model for placing the window of the wing wall. DrawWingWallVmParameter contains the path information (straight line, circular arc) and style information (cantilever, handrail, empty box) of the wing wall, and WingWallModelCurveCopyOptions indicates the copying method of the wing wall. Through the effective realization of the above functions, a complete and accurate overall model of the sluice gate can finally be flexibly pieced together, thus greatly improving the efficiency and accuracy of water conservancy project design. Figure 5 shows the specific assembly process diagram of the sluice gate.

The custom-developed template library is a set of procedures developed to solve the problems induced by model assembly, mainly through the “custom semantics and project layout scheme”, achieving the intelligent assembly of sluice gate projects so as to solve the complexity and uncertainty of the interface between models. Therefore, it can be considered that on the basis of parametric family modeling, making full use of the Revit API function and combining this with the MVVM development mode, the automatic assembly process of sluice gate project models can be realized quickly.

## 5. Conclusions

The complex process of the parametric modeling and integration of sluice gate engineering is explored in depth, and an efficient and reliable solution for sluice gate engineering design is provided through the introduction of inter-model constraints and an automatic assembly template library based on Revit API. The outcome is specifically summarized in the following two aspects:

(1) Parametric modeling steps that consider constraints between components. The building sequence is determined based on the constraints, which ensures the reasonableness of the model assembly, and moreover, it improves the model’s precision at the source and enhances the accuracy of the model’s construction.
(2) The development of a template library for sluice modeling for automated assembly. Based on the development of Revit API, a template library for the automatic assembly of each component of the sluice project was created, which greatly simplifies the process of model construction and improves work efficiency. The template library for automated modeling not only solves the cumbersome procedures and model errors induced by model assembly, but also provides a strong tool support for future water conservancy project design. The template library is not only a revolutionary simplification of the traditional modeling process, but also a far-reaching layout for future water conservancy engineering design practice.

## Figures and Tables

**Table 1:** Main functions or classes of template library.

Name	Clarification
TemplateArrangeView	Template layout view
TemplateArrangeVm	Template layout view model
DrawingCanvas	Customized drawing boards
TemplateConfigView	Template setting view
WindowPlaceBottomPlate	Placing the baseboard window
PlaceBottomPlateVm	Placement of the base plate view model
IPlaceLockChamberBottomPlate	Docking board interface
WindowPlaceWingWall	Placement of wing wall windows
PlaceWingWallVm	Placement of wing wall view model

**Table 3:** A parameterized family breakdown of the Shiyan sluice gate.

Triangular shear model	Triangular stiffening rib based on circular arc (right)	Linear cavities	Center pier
Pedestrian bridge	Triangular stiffening rib based on circular arc (left)	Circular toothed curb	Center pier trim
Gate chamber base plate	Triangular stiffener rib based on straight line (right)	Concrete berm (upstream)	Working bridge center pier
Arc handrail	Triangular stiffening rib based on straight line (left)	Concrete berm (downstream)	Side piers
Waveguard	De-stressing pool	Elevation gate	Side pier trim
Circular wing wall	Cow leg	Wing wall shear model	Working bridge side piers
Circular wing wall bedding	Straight line bedding toothed cantilever	Footbridge	Upstream
Rounded corners	Straight line handrail	Side pedestrian bridge	Downstream
Straight corners	Linear waveguard	Drainage holes (surface-based, cross-alignment)	Rounded steel parapet (right)
Straight wing walls	Chamfering of linear waveguards	Straight steel parapet	Rounded steel parapet (left)
Straight wing wall bedding	Concrete bottom protection		

## Data Availability

Data are contained within the article.
